# Chitosan-capped gold nanoparticles for selective and colorimetric sensing of heparin

**DOI:** 10.1007/s11051-013-1930-9

**Published:** 2013-08-25

**Authors:** Zhanguang Chen, Zhen Wang, Xi Chen, Haixiong Xu, Jinbin Liu

**Affiliations:** 1 Department of Chemistry, Shantou University, Shantou, 515063 China; 2 Shantou Central Hospital, Affiliated Shantou Hospital of SUN YAT-SEN University, Shantou, 515031 China; 3 Department of Chemistry, University of Texasat Dallas, Richardson, TX 75080 USA

**Keywords:** Gold nanoparticles, Resonance light scattering, Chitosan, Heparin, Colorimetric detection

## Abstract

In this contribution, novel chitosan-stabilized gold nanoparticles (AuNPs) were prepared by mixing chitosan with citrate-reductive AuNPs under appropriate conditions. The as-prepared chitosan-stabilized AuNPs were positively charged and highly stably dispersed in aqueous solution. They exhibited weak resonance light scattering (RLS) intensity and a wine red color. In addition, the chitosan-stabilized AuNPs were successfully utilized as novel sensitive probes for the detection of heparin for the first time. It was found that the addition of heparin induced a strong increase of RLS intensity for AuNPs and the color change from red to blue. The increase in RLS intensity and the color change of chitosan-stabilized AuNPs caused by heparin allowed the sensitive detection of heparin in the range of 0.2–60 μM (~6.7 U/mL). The detection limit for heparin is 0.8 μM at a signal-to-noise ratio of 3. The present sensor for heparin detection possessed a low detection limit and wide linear range. Additionally, the proposed method was also applied to the detection of heparin in biological media with satisfactory results.

**Electronic supplementary material:**

The online version of this article (doi:10.1007/s11051-013-1930-9) contains supplementary material, which is available to authorized users.

## Introduction

Heparin, known as the most negatively charged biological macromolecule (Rabenstein 2002; Althaus et al. 2013; Linhardt and Toida 2004; Whitelock and Iozzo 2005), is a highly sulfated linear glycoaminoglycan consisting of repeating units of 1→4-linked pyranosyluronic acid and 2-amino-2-deoxyglucopyranose residues. Heparin plays an important role in the regulation of various normal physiological and pathological processes such as cell growth and differentiation, inflammation, immune defense, lipid transport and metabolism, and blood coagulation (Althaus et al. 2013; Whitelock and Iozzo 2005; Mackman 2008; Williams and Davies 2001). Heparin has been widely used as a major anticoagulant with about half a billion doses applied annually (Szelke et al. 2010). However, heparin overdose can induce some complications such as hemorrhages and heparin-induced thrombocytopenia (Wallis et al. 1998; Koster et al. 2013). The proper therapeutic dosing concentration range of heparin is 2–8 U/mL (17–67 μM) during cardiovascular surgery and 0.2–1.2 U/mL (1.7–10 μM) in the postoperation period and long-term therapy (Zhan et al. 2010). Therefore, close monitoring and quantification of heparin in serum are of vital importance for regulation of the physiological process and for clinical applications during surgery and the postoperative therapy period.

Many assays for heparin quantification or detection have been established. Traditional laboratory assays, such as activated coagulation time and activated partial thromboplastin time, are indirect and not sufficiently reliable, accurate, or amenable to clinical settings (Murray et al. 1997; Fu et al. 2012a, b). Thus, the development of new methods with high accuracy and reliability is very much desirable.

Recently, a variety of fluorescent methods have been reported for heparin sensing (Wright et al. 2005; Mecca et al. 2006; Sun et al. 2007; Sauceda et al. 2007; Dai et al. 2011; Yan and Wang 2011). Some cationic chromophores, such as tripodal boronic acids (Wright et al. 2005), polycationic calix[8]arenas (Mecca et al. 2006), and a chromophore-tethered flexible copolymer (Sun et al. 2007), have been reported as heparin sensors. Most of these assays adopt fluorescence quenching as the signal output, which is undesirable for heparin detection because of large environment effects or low sensitivity. Subsequently, a peptide-based sensor was reported to show fluorescence increase upon interaction with heparin (Sauceda et al. 2007). Otherwise, a reversed-phase ion pair high-performance liquid chromatography (RPIP-HPLC) was developed for the separation of heparin using a C_18_ column (Patel et al. 2009). However, these methods reported in the literature are usually restricted to complex instruments, are time-consuming, or have low sensitivity. Therefore, development of simple and reliable methods for heparin detection has attracted immense interest.

Resonance light scattering (RLS), an elastic scattering, occurs when the incident beam is close to its molecular absorption bands. The light scattering signals can be easily detected by synchronously scanning both the excitation and emission monochromators with a conventional spectrofluorometer (Pasternack et al. 1993; Pasternack et al. 1995; Brar and Verma 2011; Ling et al. 2009; Ling et al. 2007). To date, RLS has been applied to the study and determination of nucleic acids (Huang et al. 1997), adenine (Xu et al. 2012), amino acid (Chen et al. 2012), and screening of anticancer drugs (Chen et al. 2010; Chen et al. 2011). With the development of nanoscience and nanotechnology, gold nanoparticles (AuNPs) based on RLS methods have been attracting enormous attention in biomolecule detection, since the light scattering ability of AuNPs is about four orders of magnitude stronger than that of a fluorescent dye (Li and Li 2009). Otherwise, AuNPs have been extensity used as a colorimetric probe for the detection of breast cancer biomarker and bacteria (Carey et al. 2011; Laurieri et al. 2010). However, the AuNPs were used in the above-mentioned studies prepared by citrate-reduced AuCl_4_^−^ without any functionalization. To date, a variety of methods have been developed to generate monodisperse AuNPs (Schrofel et al. 2011; Moreno-Alvarez et al. 2010; English and Waclawik 2012; Singh et al. 2010). Recently, AuNPs synthesized with polymers as both the reducing and stabilizing agent were reported to improve their stability and biocompatibility (Sardar et al. 2007). Chitosan, the second most naturally abundant polysaccharide, consists of glucosamine and N-acetyl glucosamine units linked together by β-1,4-glucosidic bonds. Due to its biodegradable, biocompatible, and non-toxic characteristics, chitosan is utilized in many areas. However, to the best of our knowledge, there is no report on the use of chitosan as a stabilizing agent to prepare AuNPs. The major advantage for chitosan as a stabilizing agent is that it can be used to tailor the nanocomposite properties and also to provide long-term stability to the nanoparticles by preventing particle agglomeration.

In this study, novel chitosan-stabilized AuNPs were prepared by mixing citrate-reductant AuNPs with chitosan under appropriate conditions. RLS studies indicated that chitosan-stabilized AuNPs exhibited weak RLS intensity in solution. However, the weak RLS intensity of chitosan-stabilized AuNPs was increased by the addition of a trace amount of heparin, and the RLS intensity of chitosan-stabilized AuNPs increased with the increasing concentrations of heparin. Based on these phenomena, a RLS method for the determination of heparin was developed in this contribution, which was simple, sensitive, and accurate.

## Materials and methods

### Materials and reagents

Chloroauric acid (HAuCl_4_) was purchased from the Sinopharm Group Chemical Reagent Co., Ltd. Reagent Inc. Chitosan (deacetylation degree of 0.95 and molecular weight of ~20,000 Da) was purchased from Beijing Chemical Reagent Co. (Beijing, China). Sodium citrate was purchased from Beijing Chemical Reagent Company (Beijing, China). Hyaluronic acid (HA) salt and chondroitin 4-sulfate were obtained from Streptococcus equi (BioChemika). DNA was provided by Shanghai Shenggong Co. (Shanghai, China). Heparin sodium salt was obtained from Aladdin Chemistry Co. Ltd (185 U/mg, Shanghai, China). Heparin stock solution (1.00 mg/mL) was prepared by dissolving 0.1000 g of heparin sodium reagent in water and diluting to the mark in a 100-mL calibrated flask. The working solution was further diluted with water. All other solvents and reagents in this investigation were of analytical grade and used without further purification. The human serum samples were obtained from healthy people in the Medical College, Shantou University (Shantou, China). The Britton–Robin (BR) buffer solution was used to control the acidity of the solution, which was made up of 0.04 mol/L phosphoric acid, 0.04 mol/L acetic acid, 0.04 mol/L boric acid, and 0.2 mol/L sodium hydroxide.

### Apparatus

The RLS spectra were measured on a LS-55 fluorescence spectrophotometer (PerkinElmer, USA) equipped with a 1 × 1 cm quartz cuvette at room temperature. A N5PCS submicron particles’ size analyzer (Beckman coulter, Miami, USA) was used to detect the size of the aggregation species in solution on the basis of the dynamic light scattering (DLS) principle. All absorption spectra were recorded on a Lambda 950 UV–Vis Spectrophotometer (PerkinElmer, USA) at room temperature. The photographs were taken with a Cannon SX230 digital camera. All pH measurements were made with a DELTA 320s acidity meter (Mettler-Toledo Instruments Co. Ltd., Shanghai, China).

### Experimental procedure

#### Preparation of gold nanoparticles (AuNPs)

All glassware used in the following procedure was cleaned in a bath of freshly prepared chromate washings, rinsed thoroughly in water, and dried in air. AuNPs were prepared according to the published protocol (Huang et al. 2005). Briefly, after boiling 50 mL of 0.01 % HAuCl_4_ solution, 3.5 mL of 1.0 % trisodium citrate solution was quickly added with vigorous stirring. The mixed solution was boiled for 10 min and further stirred for 15 min. The solution was naturally cooled to room temperature after moving away from the heater, and then diluted to 100 mL. The molar concentration of the prepared AuNPs was 15 nM according to Beer’s law using an extinction coefficient of 2.7 × 10^8^ M^−1^cm^−1^ (Haiss et al. 2007).

#### RLS spectra

Briefly, 40 μL of 10 μM chitosan was firstly added into a colorimetric tube and 400 μL of 15 nM AuNPs was then pipetted into the solution. The solution was vortex mixed and incubated for 10 min at room temperature. Then, 0.5 mL pH 6.0 BR buffer solution was added into the mixture. Next, appropriate amount of water and heparin were added into the mixture. The mixture was then vortex mixed thoroughly and kept at room temperature for another 10 min. After that, the mixture was transferred for RLS spectra. The RLS spectra were obtained by scanning synchronously the excitation and emission monochromators of the spectrofluorometer from 200 to 700 nm with Δλ = 0. Both the excitation and emission slit widths were kept at 10.0 nm. The enhanced RLS intensity of chitosan-AuNPs was represented as ΔI_RLS_ = I_RLS_ − ${\text{I}}_{\text{RLS}}^{0}$, and I_RLS_ and ${\text{I}}_{\text{RLS}}^{0}$ were RLS intensities of the chitosan-AuNPs with and without heparin, respectively.

#### UV–Vis spectra

UV–Vis absorbance spectra were obtained by a Lambda950 UV–Vis spectrophotometer (Perkin Elmer, USA) equipped with a quartz microcolorimetric vessel of 1-cm path length. In the UV–Vis assay, the preparation of solutions was the same as in the RLS spectra method. And, the scanning range was from 200 to 800 nm.

#### Detect heparin in diluted human serum

The similar detection procedure was used as described in aqueous solution, except that diluted human serum was applied as reaction matrix. The diluted human serum was prepared by adding 1 mL fresh human serum into the 99 mL BR buffer solutions and mixing well. The heparin-spiked diluted human serum samples were prepared by adding different amounts of heparin into the as-prepared diluted human serum and mixing well.

## Results and discussion

### Characterization of the obtained chitosan-stabilized AuNPs

The as-prepared chitosan-stabilized AuNPs are highly dispersed in aqueous solution and have very weak RLS intensity, as shown in Figure S1. The chitosan-stabilized AuNPs exhibit a wine red color in solution. These properties of chitosan-stabilized AuNPs impart the sensor high sensitivity and colorimetric response for heparin detection.

### Characteristics of the resonance light scattering spectra

**Fig. 1:**
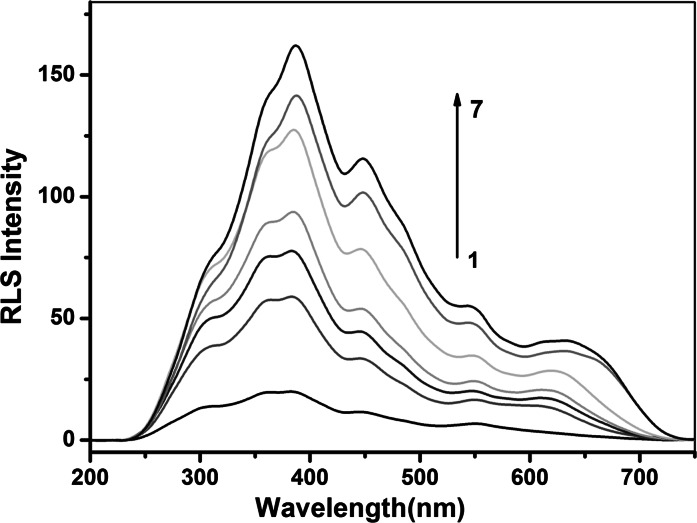
RLS spectra of AuNPs (*1*) in the absence of heparin and in the presence of (*2–7*): 2, 12, 22, 40, 44, 60 μM. Conditions: 40 μL of 10 μM chitosan, 400 μL of 15 nM AuNPs, BR (pH 6.0) buffer solution

Figure 1 shows the light scattering spectra of chitosan-stabilized AuNPs and the conjugated chitosan-stabilized AuNPs with different concentrations of heparin. It is reasonable that the diameter of chitosan-stabilized AuNPs is smaller than 1/20 of the incident wavelengths in the scan range such that the signal would only come from RLS (Miller 1978). As Fig. 1 shows, the RLS intensity of chitosan-stabilized AuNPs is very weak when they exist in aqueous solution alone. However, when the heparin was added into the solution, the RLS intensity was remarkably enhanced. Figure 1 also suggests that the enhanced RLS intensities continuously increase with gradual increasing of the heparin concentration, while the spectral profile is maintained almost the same. These situations contribute to the fact of the aggregation of chitosan-stabilized AuNPs after addition of heparin. To compare the light scattering spectra of chitosan-stabilized AuNPs with that of chitosan-stabilized AuNPs-heparin conjugates, it can be concluded that the assembly of chitosan-stabilized AuNPs results in great enhancement of RLS intensity of chitosan-stabilized AuNPs. On the other hand, the assembly of chitosan-stabilized AuNPs via heparin leads to the size increase of scatters, which results in the RLS enhancement.

### Sensor operation principle

**Scheme 1:**
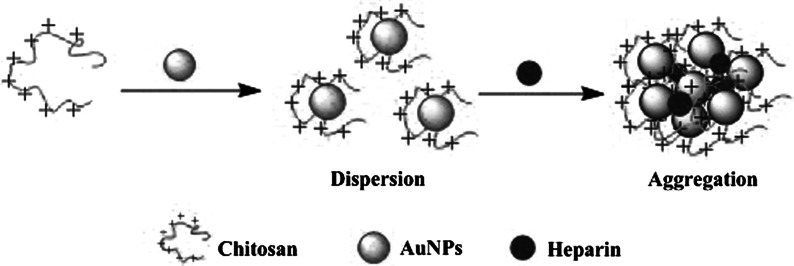
Heparin induced the aggregation of chitosan-stabilized AuNPs resulting in colorimetric responses

Scheme 1 illustrates the analytical process for detecting heparin. The synthesized AuNPs were first mixed with chitosan at pH 6.0 BR buffer solution. Then, Chitosan could quickly adsorb onto the surface of AuNPs. Because of the –NH_3_^+^ group of chitosan, the chitosan-stabilized AuNPs are positively charged. The chitosan-stabilized AuNP solution is stabilized against aggregation due to the positive capping agent’s electrostatic repulsion between AuNPs. Next, the functionalized chitosan-AuNPs were induced to aggregate in the presence of heparin through electrostatic attraction between positively charged AuNPs and polyanionic heparin. Importantly, the RLS intensity of the system would increase with the aggregation of chitosan-stabilized AuNPs. Moreover, it also causes a rapid, red-to-blue color change. We just make use of this property to design our sensor for heparin.

### Colorimetric detection of heparin

**Fig. 2:**
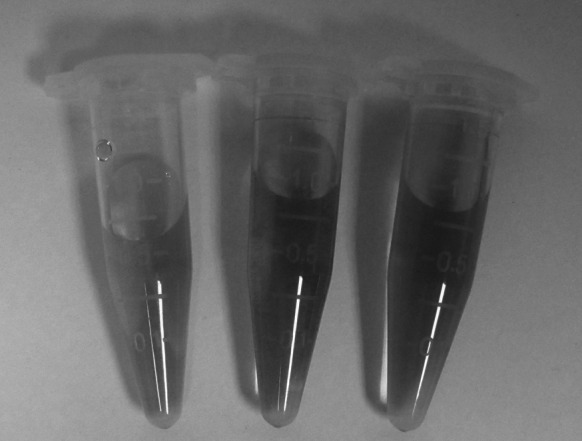
Photographs of a solution of **a** AuNPs, **b** AuNPs + heparin (40 μM), **c** AuNPs + heparin (100 μM). Experimental conditions: 40 μL of 10 μM chitosan, 400 μL of 15 nM AuNPs, BR (pH 6.0) buffer solution

To evaluate the sensitivity of heparin depending on the colorimetric assay under the optimized detection conditions, the color changes of the chitosan-stabilized AuNPs solution were recorded by a digital camera. Figure 2 shows the color changes in chitosan-stabilized AuNPs after the addition of different concentrations of heparin. As the concentration of heparin increased, the color of chitosan-stabilized AuNP solution changed from red to purple, and blue, which could be easily observed by the naked eye. Such color change can be easily used as a quantitative assay for heparin.

### Validation test

**Fig. 3:**
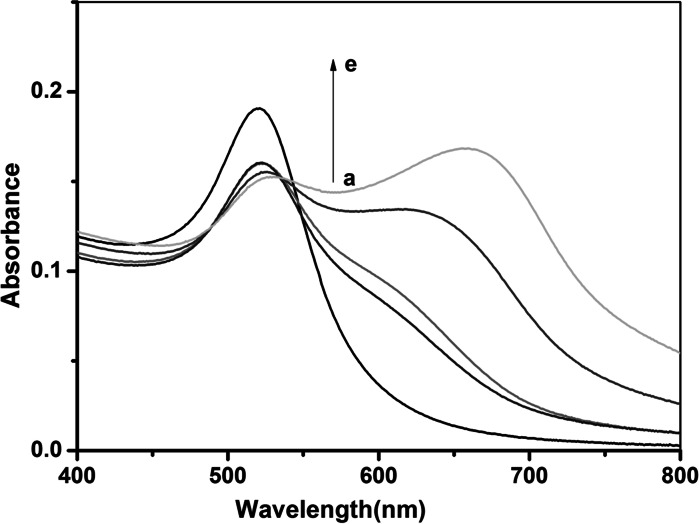
UV–Vis absorption spectra of *a* AuNPs and in the presence of different concentration of heparin, *b–e*: 2, 12, 40, 60 μM. Experimental conditions: 40 μL of 10 μM chitosan, 400 μL of 15 nM AuNPs, BR (pH 6.0) buffer solution

The RLS enhancement of chitosan-stabilized AuNPs in the presence of heparin should be due to the aggregation of chitosan-stabilized AuNPs induced by the electrostatic interactions between chitosan-stabilized AuNPs and polyanionic heparin. In order to demonstrate the conjecture above, we performed a series of investigations. It was found that the particle sizes of the aggregated species, as disclosed by the DLS measurements, which could well reflect the size changes of microparticles in solution based on scattering signals (Liu et al. 2009; Long et al. 2007), increase with increasing heparin concentration (Figure S2). Identical to the DLS analysis, we also observed the heparin-induced state change of dispersion/aggregation of chitosan-stabilized AuNPs by UV–Vis spectra. The result is shown in Fig. 3. Note that in the absence of heparin, the chitosan-stabilized AuNPs have one characteristic peak centered at 520 nm, which is ascribed to the surface plasmon resonance absorption corresponding to the dispersed AuNPs (Grabar et al. 1995). When chitosan-stabilized AuNPs aggregate and the interparticle distance in these aggregates decreases to less than approximately the average particle diameter, it results in the shift of the absorption band to longer wavelengths and the color of the aggregates turns blue, because of electric dipole–dipole interaction and coupling between the plasmons of neighboring particles in the formed aggregates (Kreibig and Genzel 1985). As shown in Figure S3, a new absorption peak of AuNPs appears at 660 nm when increased heparin is mixed with chitosan-stabilized AuNPs. Thus, it confirmed that the principle of the sensor is reasonable from the experimental results above.

### Effect of chitosan concentration

Mixing a suitable ratio of chitosan with AuNPs can make the probe more effective, enhance RLS intensity, and make the heparin detection more accurate. A sufficient amount of chitosan, which can make AuNPs completely dispersed, is necessary to obtain a minimal reagent RLS blank. On the contrary, the amount of chitosan cannot be excessive or the accuracy and the sensitivity will be ultimately affected. Therefore, the ratio of the amount of chitosan and AuNPs was studied. Figure 3 shows the different RLS intensities based on varying chitosan-AuNPs ratios. In the tridimensional data, we could see that the best volume ratio was AuNPs:chitosan = 10:1. Since the AuNP concentration was 15 nM and the chitosan was 10 μM, the best ratio of the chitosan to AuNPs was 66.7 (nM/nM). Thus, in the preparation of chitosan-AuNPs conjugates, 40 μL chitosan was added into 400 μL AuNP solution to make the detection accurate and sensitive.

### Kinetics of RLS enhancement of chitosan-AuNPs conjugates in the presence of different amounts of heparin

**Fig. 4:**
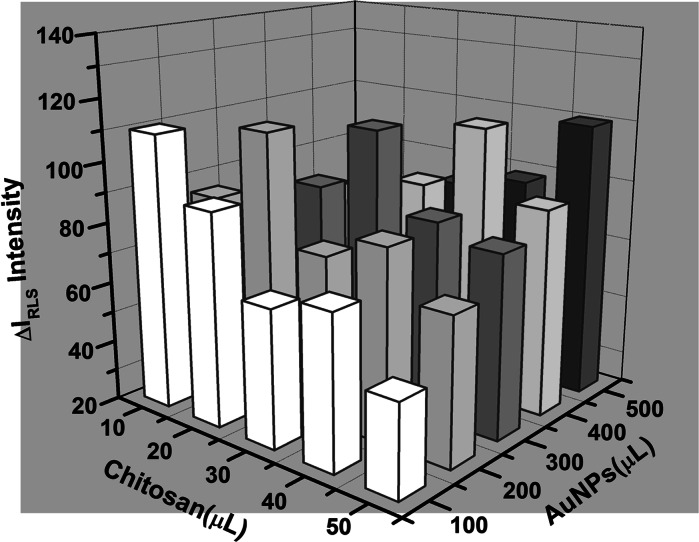
Effect of the ratio of chitosan and AuNPs on the detection of 44 μM heparin. Conditions: BR (pH 6.0) buffer solution

Since the combination of chitosan-AuNPs and heparin and the aggregation of AuNPs in the detection system were all dynamic processes, the relationship between RLS intensity and time of the chitosan-AuNP conjugates in the presence of different amounts of heparin was studied and the result is shown in Fig. 4. It was clear that RLS intensity was enhanced gradually with time and kept constant after 13 min when heparin was 2 μM in the chitosan-AuNP system. Subsequently, with the addition of heparin, the RLS intensity of this system could be stable more quickly. In order to achieve the best results of determination, 15 min was selected as the reaction time in the RLS detection stage.

### Effect of pH and temperature

In order to select a sensitive, simple, and practical assay for heparin, the RLS detection conditions were optimized in this study. The effects of pH and temperature were investigated in our current work. The effect of pH on the response of the chitosan-stabilized AuNP probe was investigated at a pH range from 2.5 to 8.5 (shown in Figure S3). The RLS intensity versus media pH was obtained by adjusting media pH with different concentration ratios of acid to base and fixing the heparin concentration at 44 μM. The experimental results showed that at low pH, the RLS intensity of the system was obviously increased by adding heparin, but when some other substances (such as HA) were added into the system, the RLS intensity was also greatly increased (not shown in Figure S3), which is due to the pKa of –NH_3_^+^ group that is 10.75. The amine group of chitosan is protonated at low pH (pH <6.5), so the chitosan-stabilized AuNPs are positively charged, which leads to the efficient electrostatic interaction with the negative-charged substances. These results are understandable by considering the fact that chitosan-stabilized AuNPs will provide more positive charge for interaction with HA at very low pH, leading to aggregation of chitosan-stabilized AuNPs, while chitosan-stabilized AuNPs can only offer limited positive charges for interaction with heparin at pH 6.0, and as a result the interference of these substances could be ignored. Thus, we could control the media pH in order that the heparin could selectively bind chitosan-stabilized AuNPs. Finally, pH 6.0 BR buffer solution was chosen as the reaction media. Binding temperature influenced the response of the sensor. In the range of 20–45 °C, the ΔI_RLS_ decreased with increasing temperature. The possible reason is that high temperature could induce self-aggregation of AuNPs and weaken the electrostatic interaction of chitosan-stabilized AuNPs and heparin. Hence, room temperature (ca. 20 °C) was used for the detection temperature.

### Specificity of the sensor

To demonstrate the specificity for the detection of heparin on the chitosan-AuNPs based on the colorimetric assay, competition experiments for selectivity to other molecules with similar molecular structures to heparin, and to commonly coexistent physiological level species, were further carried out. As shown in Figure S4, in the presence of HA and Chs, the RLS intensity of chitosan-stabilized AuNPs had a slight increase compared to that given by the blank; interestingly, heparin led to a remarkable RLS increase at an identical concentration with HA and Chs. This was just due to the low charge density per repeat unit of HA and Chs, with only one carboxyl group and two groups of sulfate and carboxylate moieties, respectively. However, heparin possesses three sulfate groups and one carboxylate per repeat unit (Rabenstein 2002). Thus, the electrostatic attraction between chitosan-AuNPs and heparin was significantly stronger than that between chitosan-stabilized AuNPs-HA and chitosan-stabilized AuNPs-Chs. Calf thymus DNA was used as a model to investigate the effect of other polyanionic substances. The experimental results showed that 50 ng/mL of DNA (plasma DNA normal level) did not interfere with the detection of heparin. (Figure S5) In addition, the general presence of physiological species of Na^+^, K^+^, Mg^2+^, Ca^2+^, Cl^−^, glucose, and cysteine was also found not to affect the detection of heparin. It demonstrated that the developed assay provided attractive specificity toward heparin.

### Quantitative detection of heparin

**Fig. 5:**
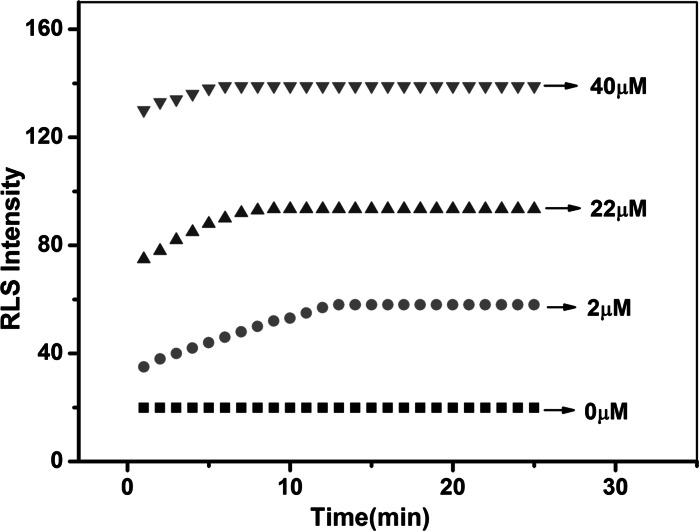
Kinetics of RLS enhancement of AuNPs-chitosan conjugates in the presence of different amounts of heparin. Conditions: 40 μL of 10 μM chitosan, 400 μL of 15 nM AuNPs, BR (pH 6.0) buffer solution

**Fig. 6:**
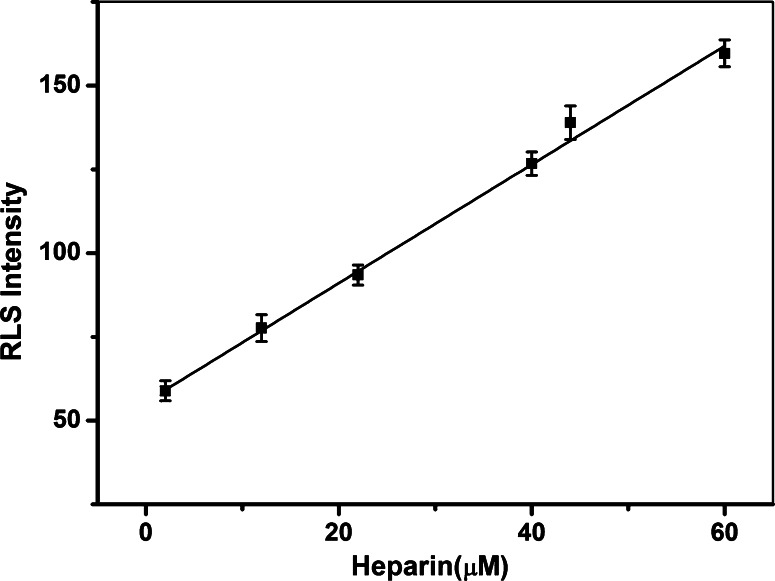
Plots of the RLS intensity versus heparin concentration. Experimental conditions: 40 μL of 10 μM chitosan, 400 μL of 15 nM AuNPs, BR (pH 6.0) buffer solution

Under the optimized conditions, the RLS spectra of chitosan-stabilized AuNPs in the presence of heparin with different concentrations were recorded. Figure 1 illustrates that the RLS spectra of chitosan-stabilized AuNPs gradually increased with increasing concentration of heparin. The RLS intensity of chitosan-stabilized AuNPs exhibited a good linear correlation to the concentration of heparin in the range of 0.2–60 μM (*R* = 0.9981) (Fig. 5). The standard regression equation is *I* = 1.77C + 55.61. A series of 5 repetitive measurements of 12 μM heparin were used for estimating the precision, and the relative standard deviation was 1.3 %, showing that the strategy had good reproducibility. Based on 3σ/*s* (σ is the standard deviation of the blank measurements and *s* is the sensitivity of the calibration graph), the detection limit of heparin was calculated to be 0.8 μM. It is comparable to or even better than previous reports for heparin detection (Sauceda et al. 2007). The result implied that this method showed a highly sensitive response to heparin (Fig. 6).

### Real sample analysis

The development of a selective and sensitive sensor in the physiological condition for heparin is crucial. To investigate the practical application of this method, the detection of human serum sample was carried out by the standard addition method. In the experiment, five different amounts of heparin were added into a certain amount of diluted human serum. No other pretreatment was performed. The treated human serum samples were added into the chitosan-stabilized AuNP solution and incubation for 10 min. Then, the samples were measured on the fluorescence spectrophotometer. Each sample was measured five times. The results are presented in Table S1. From the Table S1, we can see that the method had good recovery and the measured recoveries were between 103 and 96 % with less than 3.5 % RSD under the optimal conditions. The results showed that the recovery and precision of the proposed method were satisfactory. Therefore, we think our proposed method could be employed to the detection of heparin in human serum samples.

## Conclusions

In summary, a novel methodology for the detection of heparin has been developed based on the RLS increase of heparin on the RLS of chitosan-stabilized AuNP system. Our prepared chitosan-stabilized AuNPs exhibited weak RLS intensity in solution. With the addition of an increasing concentration of heparin to chitosan-stabilized AuNP solution, an obvious increase in the RLS intensity was clearly detected. In addition, the interaction between heparin and chitosan-stabilized AuNPs induced a corresponding color change from wine red to blue. Thus, the concentration of heparin could be determined with the naked eye in a certain range. Results showed that the RLS intensity was linear with the heparin concentration in the range of 0.2–60 μM (~6.7U/mL) with linear coefficients of 0.998. The detection limit was as low as 0.8 μM, which was much lower than most existing methods. The assay reported herein may find applications in research requiring rapid detection and quantification of purified heparin samples or heparin in biological media.

## Electronic supplementary material

Below is the link to the electronic supplementary material.

Supplementary material 1 (DOC 653 kb)
