# Supplementary material for: Chitosan-capped gold nanoparticles for selective and colorimetric sensing of heparin
Source: J Nanopart Res. 2013 Aug 25;15(9):1930. doi: 10.1007/s11051-013-1930-9 (PMC3782634; doi:10.1007/s11051-013-1930-9)
Supplement: Supplementary file 1 — Supplementary material 1 (DOC 653 kb) [file 11051_2013_1930_MOESM1_ESM.doc]

**Supporting Information**

**Chitosan-capped Gold Nanoparticles for Selective and Colorimetric Sensing of Heparin**

Zhanguang Chen*^,a^, Zhen Wang^a^, Xi Chen^b,a^, Haixiong Xu^b^, Jinbin Liu^c^

*^a^Department of Chemistry, Shantou University, Shantou 515063, China*

*^b^Shantou Central Hospital, Affiliated Shantou Hospital of SUN YAT-SEN University, Shantou 515031, China*

*^c^Department of Chemistry, University of Texasat Dallas, Richardson, TX75080, USA*

*Corresponding author: Department of Chemistry, Shantou University, Shantou 515063, China. E-mail address:* [*kqlu@stu.edu.cn*](mailto:kqlu@stu.edu.cn) *(Z. Chen). Tel.: +86 75482903330; fax: +86 75482902767.*


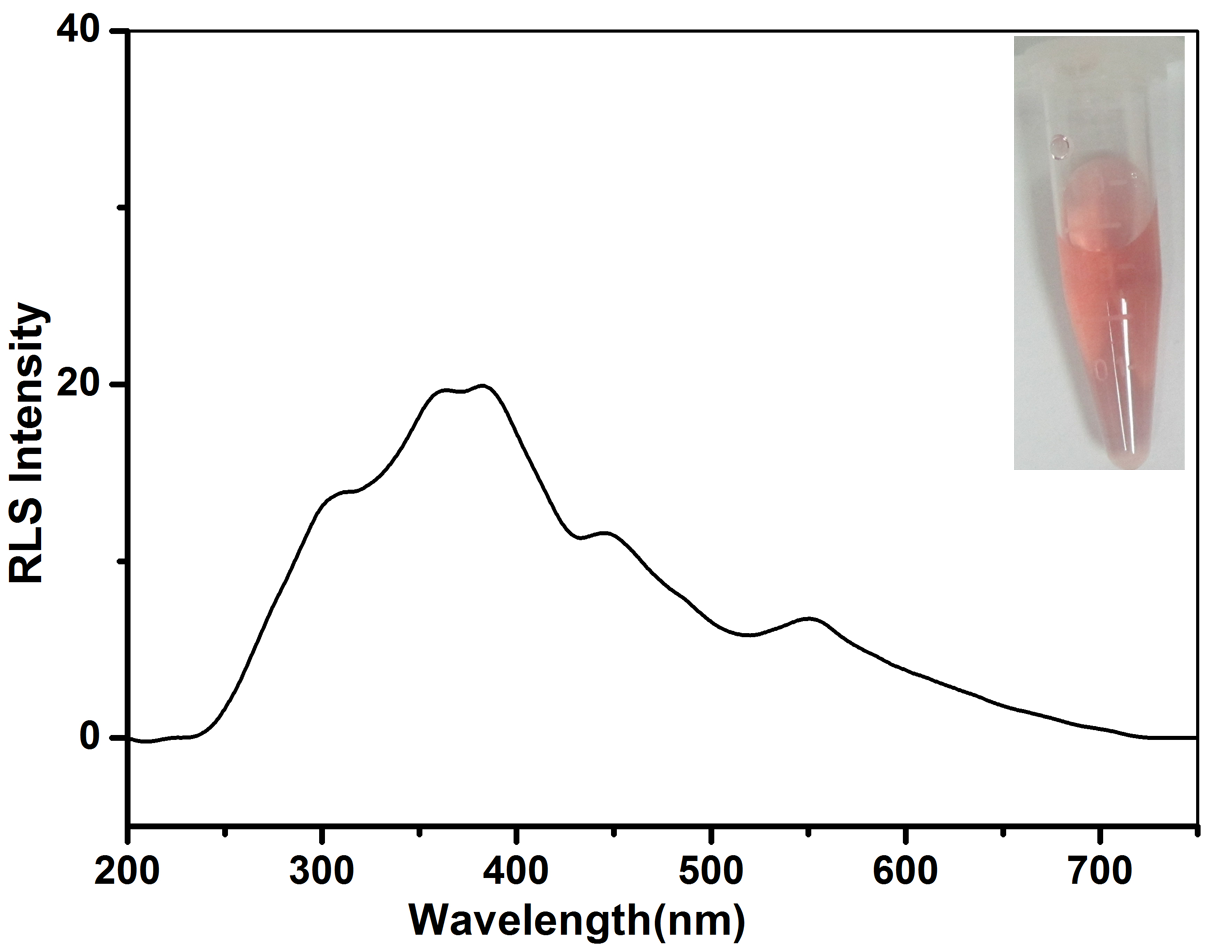


**Figure S1** The RLS intensity of chitosan-stabilized AuNPs in pH 6.0 BR buffer solution. The inset is the photograph of chitosan-stabilized AuNPs solution.


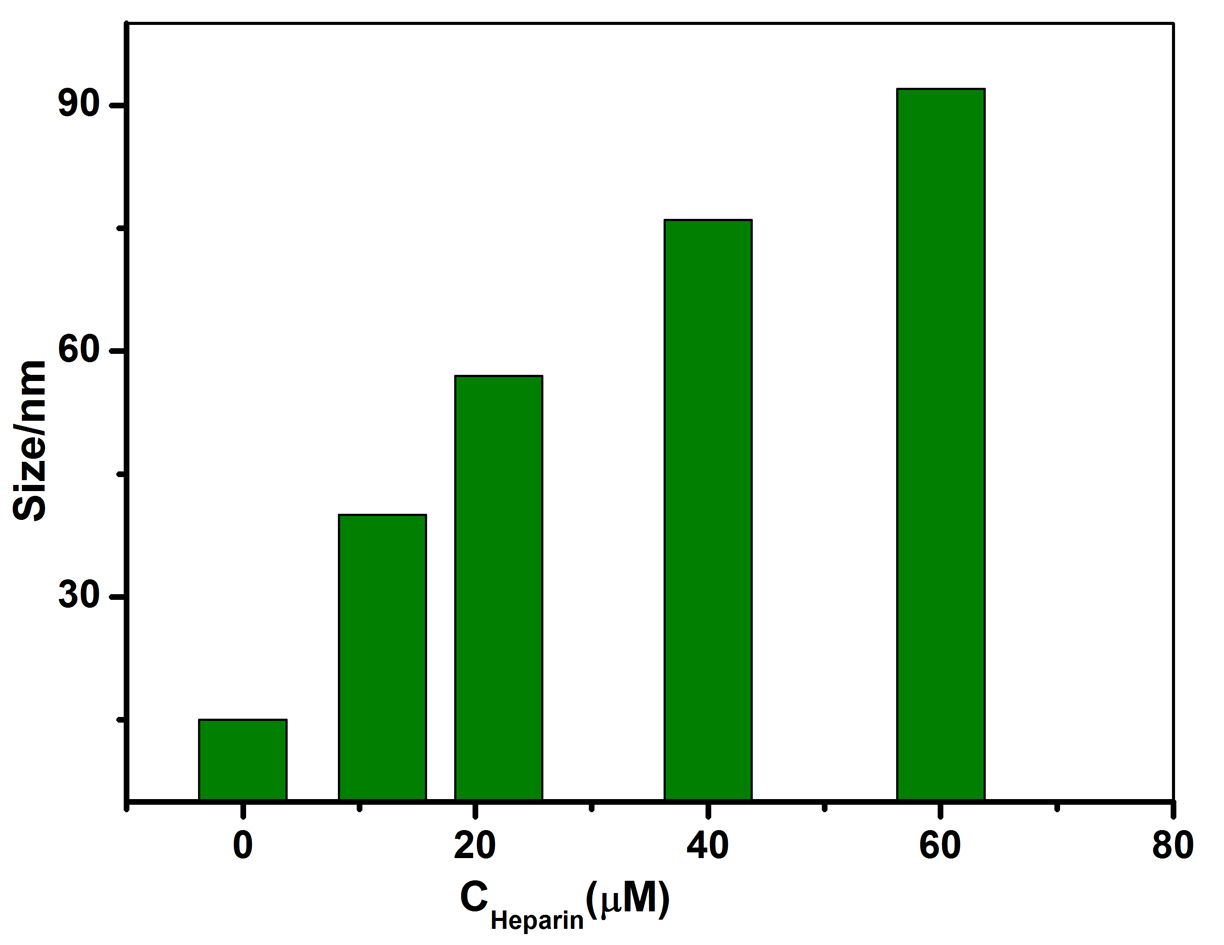


**Figure S2** DLS measurements showing the aggregation of chitosan-capped AuNPs in the presence of different concentration of heparin. Experimental conditions: 40 μL of 10 μM chitosan, 400 μL of 15 nM AuNPs, BR (pH 6.0) buffer solution.


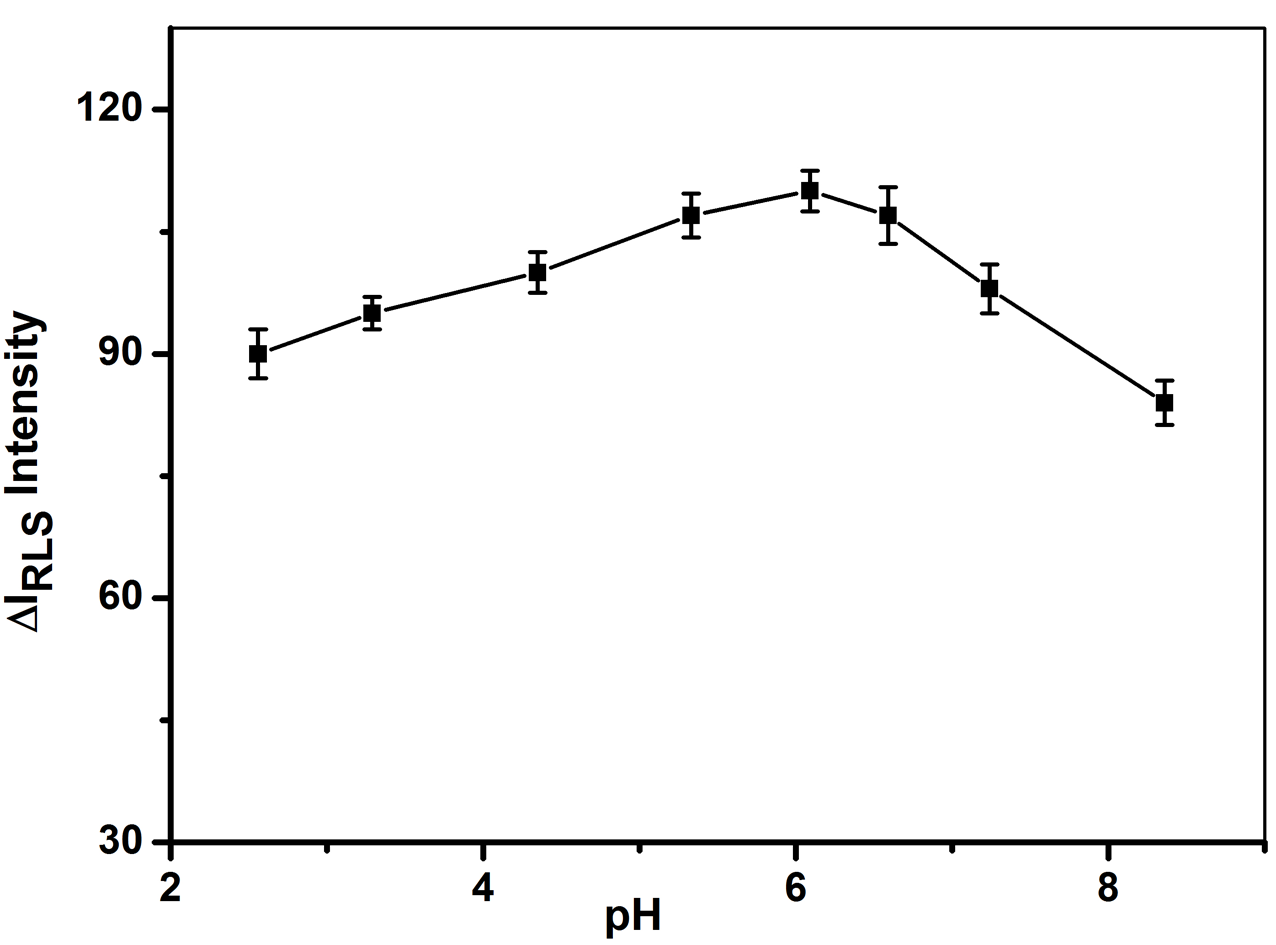


**Figure S3** Effect of pH value on the detection of 44 μM heparin. Experimental conditions: 40 μL of 10 μM chitosan, 400 μL of 15 nM AuNPs. The pH values of the solutions were controlled by BR buffer with different concentration ratio of acid to base in the pH range 2.5-8.5. Each point represents the mean ± standard deviation from five determinations.


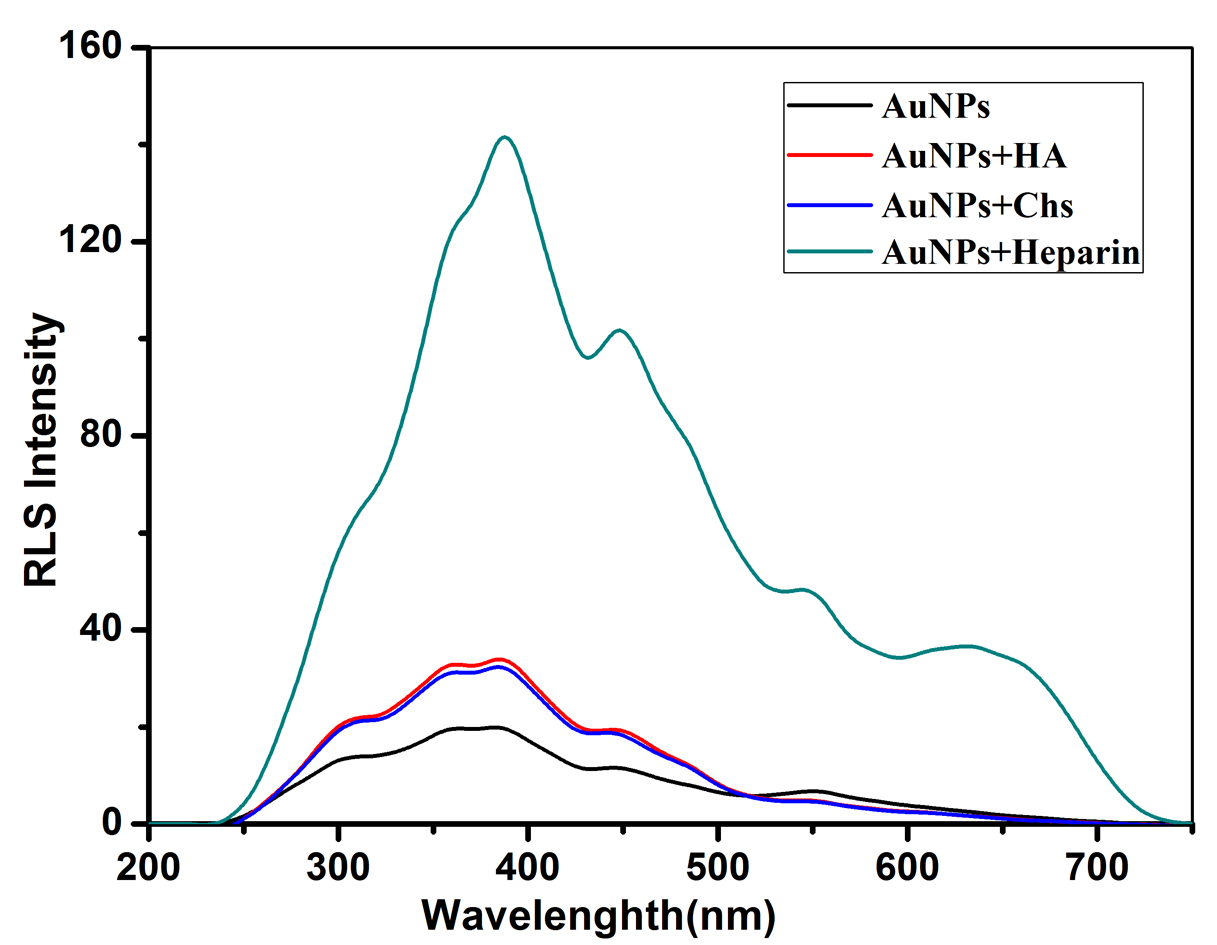


**Figure S4** RLS intensity of AuNPs (black), AuNPs +44 μM HA (red), AuNPs+44 μM Chs (blue), AuNPs+44 μM (green). Experimental condition: 40 μL of 10μM chitosan, 400 μL of 15 nM AuNPs, BR (pH 6.0) buffer solution.


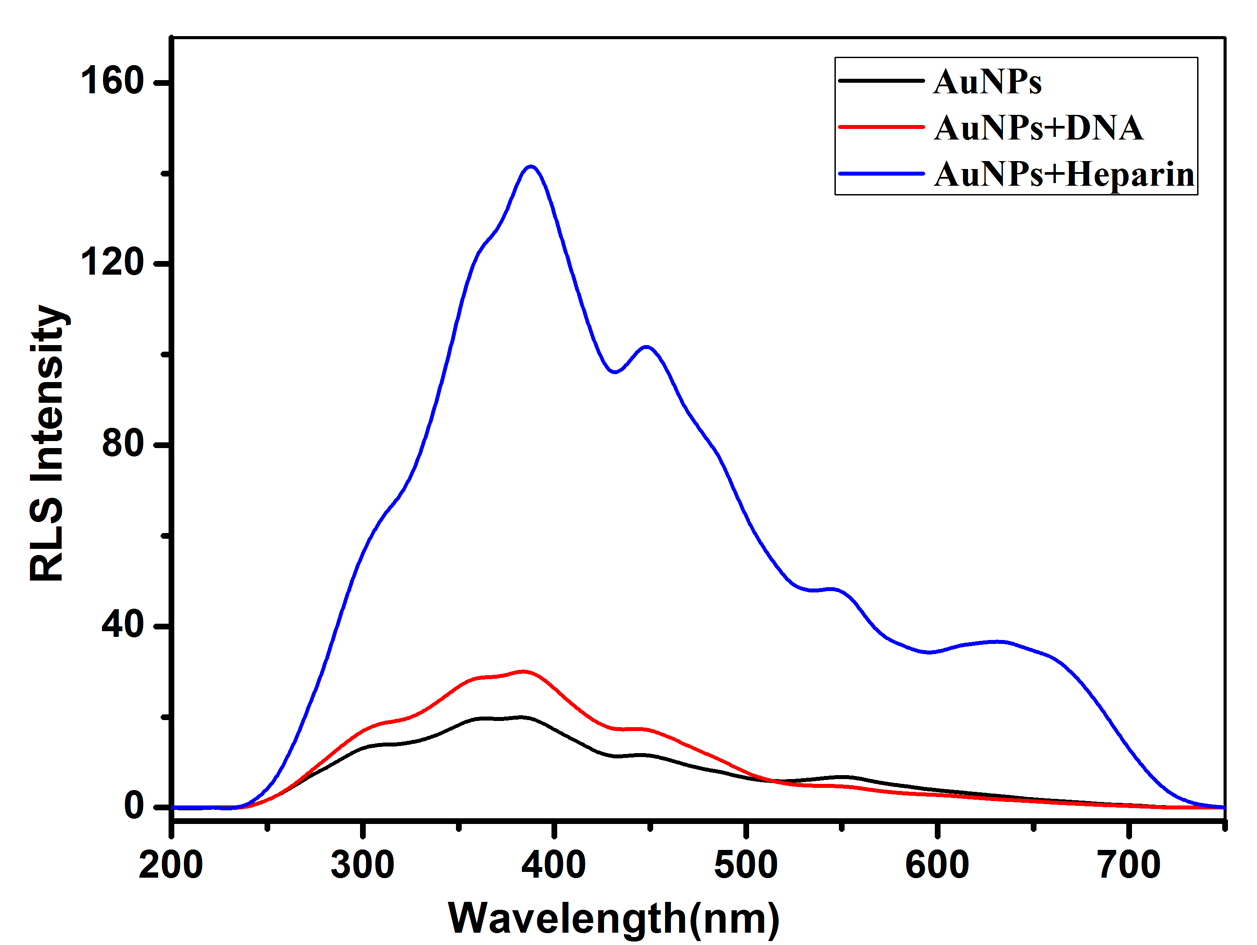


**Figure S5** RLS intensity of AuNPs (black), AuNPs + 50 ng/mL DNA (red), AuNPs+ 44 μM heparin (blue). Experimental condition: 40 μL of 10μM chitosan, 400 μL of 15 nM AuNPs, BR (pH 6.0) buffer solution.

**Table S1** Results of determination of heparin in human serum samples using the RLS method.

| Sample number | Heparin(added)  μM | Heparin(detected)^a^  μM | Recovery  (%) | RSD(%,n=5) |
| --- | --- | --- | --- | --- |
| 1  2  3  4  5 | 5  10  20  30  50 | 4.92  10.15  19.96  30.69  50.25 | 98.4  101.5  99.8  102.3  100.5 | 2.43  1.84  2.23  3.05  1.28 |

^a^The data are given as the average value obtained from five independent experiments (n=5).
